# Structural Neural Substrates of Reading the Mind in the Eyes

**DOI:** 10.3389/fnhum.2016.00151

**Published:** 2016-04-11

**Authors:** Wataru Sato, Takanori Kochiyama, Shota Uono, Reiko Sawada, Yasutaka Kubota, Sayaka Yoshimura, Motomi Toichi

**Affiliations:** ^1^Department of Neurodevelopmental Psychiatry, Habilitation and Rehabilitation, Graduate School of Medicine, Kyoto UniversitySakyo-ku, Kyoto, Japan; ^2^Brain Activity Imaging Center, Advanced Telecommunications Research Institute InternationalKyoto, Japan; ^3^Health and Medical Services Center, Shiga UniversityHikone Shiga, Japan; ^4^Faculty of Human Health Science, Graduate School of Medicine, Kyoto UniversitySakyo-ku, Kyoto, Japan; ^5^The Organization for Promoting Developmental Disorder ResearchSakyo-ku, Kyoto, Japan

**Keywords:** dorsomedial prefrontal cortex (dmPFC), reading the mind in the eyes test, structural magnetic resonance imaging (MRI), temporoparietal junction (TPJ), theory of mind (TOM), voxel-based morphometry (VBM)

## Abstract

The ability to read the minds of others in their eyes plays an important role in human adaptation to social environments. Behavioral studies have resulted in the development of a test to measure this ability (Reading the Mind in the Eyes Test, revised version; Eyes Test), and have demonstrated that this ability is consistent over time. Although functional neuroimaging studies revealed brain activation while performing the Eyes Test, the structural neural substrates supporting consistent performance on the Eyes Test remain unclear. In this study, we assessed the Eyes Test and analyzed structural magnetic resonance images using voxel-based morphometry (VBM) in healthy participants. Test performance was positively associated with the gray matter volumes of the dorsomedial prefrontal cortex, inferior parietal lobule (temporoparietal junction), and precuneus in the left hemisphere. These results suggest that the fronto-temporoparietal network structures support the consistent ability to read the mind in the eyes.

## Introduction

The ability to read the minds of others in their eyes is an indispensable ability in human social life. By inferring others’ mental states, commonly referred to as having a theory of mind (TOM; Premack and Woodruff, 1978), we can effectively understand and predict others’ behaviors, which facilitates our survival and reproductive success in social environments (Baron-Cohen, 1995). Due to this mind-reading ability, and because humans rely heavily on the eyes during face-to-face communication (Argyle and Cook, 1976), people have long believed that the “eyes are the window to the soul” (McCarty, 2011).

Behavioral studies have resulted in the development of a reliable and valid test to measure this ability: the Reading the Mind in the Eyes Test, Revised Version (hereafter, Eyes Test; Baron-Cohen et al., 2001). Because the original test was composed of only Caucasian stimuli, an Asian version of the Eyes Test was developed to provide culturally optimized stimuli; this version was subsequently confirmed to be effective (Adams et al., 2010). A previous study reported that the reliability of the Eyes Test was high, even over a 1-year period (Fernández-Abascal et al., 2013). Performance on this test was shown to correlate with an empathic personality (Lawrence et al., 2004; Voracek and Dressler, 2006). Behavioral genetics studies revealed a genetic influence on performance on the Eyes Test (Rodrigues et al., 2009; Warrier et al., 2013; Gong et al., 2014). Studies in clinical samples showed that performance on the test was worse in individuals with genetically influenced neuropsychiatric disorders, such as autism spectrum disorders (e.g., Baron-Cohen et al., 1997) and schizophrenia (e.g., Irani et al., 2006; for a review, see Chung et al., 2014). These data suggest that reading the mind in the eyes is a measurable and consistent cognitive ability.

Several functional neuroimaging studies using magnetic resonance imaging (MRI) have explored brain activation patterns while individuals perform the Eyes Test (Baron-Cohen et al., 1999; Platek et al., 2004; Adams et al., 2010; Castelli et al., 2010; Mascaro et al., 2013; for a review, see Schurz et al., 2014). These studies consistently indicated that the dorsomedial prefrontal cortex (dmPFC) and temporoparietal junction (TPJ; the posterior superior temporal gyrus and inferior parietal lobule) are activated during the test. This result is consistent with the broader literature suggesting that the dmPFC and TPJ are generally activated while individuals perform TOM tasks, such as observing cartoons in which mental state reasoning is necessary to understand the meaning (e.g., Gallagher et al., 2000; for reviews, see Van Overwalle and Baetens, 2009; Schurz et al., 2014). Furthermore, although results are not consistent across studies, several other brain regions are also reported to be activated during the Eyes Test, including the amygdala (Baron-Cohen et al., 1999; Castelli et al., 2010; Mascaro et al., 2013), inferior frontal gyrus (IFG; Baron-Cohen et al., 1999; Adams et al., 2010; Castelli et al., 2010; Mascaro et al., 2013), and precuneus (Baron-Cohen et al., 1999; Castelli et al., 2010). Together, the involvement of these multiple brain regions may constitute a widespread neural network for inferring the mental states of other individuals via the eye region (cf. Frith and Frith, 2003; Van Overwalle and Baetens, 2009; Schurz et al., 2014).

However, the structural neural substrates that support an individual’s consistent ability on the Eyes Test remain unclear. Based on functional neuroimaging data, in addition to the general correspondence between brain activation and structure (Richardson and Price, 2009), one can speculate that structures in the aforementioned brain regions may be related to this ability. To the best of our knowledge, one structural MRI study has investigated this issue, and it reported supportive but inconclusive findings (Hirao et al., 2008). The researchers assessed the performance of Japanese schizophrenic and normal participants on the original Eyes Test, and analyzed structural MRI using voxel-based morphometry (VBM). The schizophrenia group was the main group of interest; therefore, they first analyzed group differences in voxel values. Then, cluster values detected by the group comparison were analyzed, and a positive correlation was found between the test scores and the gray matter volumes in the dmPFC in the normal group. However, because of the nature of their data analysis, whether the Eyes Test score could be associated with voxel-level gray matter volumes in the dmPFC or certain other brain regions in normal participants remains unclear. Furthermore, no information was provided regarding the white matter correlates of the Eyes Test.

In this study, we administered the Eyes Test to normal participants and acquired structural MRI. We tested Japanese participants using the Asian version of the test (Adams et al., 2010). We analyzed the association between test scores and regional volumes of the gray and white matter using VBM. We predicted that test scores would be positively associated with gray and white matter volumes in the brain regions, such as the dmPFC and TPJ, which were reported to be activated during performance of the Eyes Test as described above.

## Materials and Methods

### Participants

The study included 51 volunteers (26 females; mean ± *SD* age, 22.5 ± 4.5 years). All participants were native Japanese. All participants were right-handed, as assessed by the Edinburgh Handedness Inventory (Oldfield, 1971). A psychiatrist or psychologist administered a short structured diagnostic interview using the Mini-International Neuropsychiatric Interview (Sheehan et al., 1998); no neuropsychiatric problems were detected in any of the participants. After a detailed explanation of the experimental procedure, all participants provided written informed consent for participation. Our study was approved by the local ethics committee of the Primate Research Institute, Kyoto University. The study was also conducted in accordance with the Declaration of Helsinki.

### Task

The Asian version of the Eyes Test (Adams et al., 2010) was used to measure the ability to read the mind in the eyes. The test consisted of 36 photographs depicting only the eye region of Asian people. Four mental state terms (one target and three foils) accompanied each stimulus presented at each corner of the photograph.

The task was controlled by SuperLab Pro 2.0 (Cedrus, San Pedro, CA, USA), implemented on a Windows computer (HP Z200 SFF; Hewlett-Packard, Tokyo, Japan). The stimuli were presented on a 19” CRT monitor (HM903D-A; Iiyama, Tokyo, Japan). The photographs subtended a visual angle of 12.0° horizontally × 4.8° vertically.

### MRI Acquisition

Image scanning was performed on a 3-T scanning system (MAGNETOM Trio, A Tim System; Siemens, Malvern, PA, USA) at the ATR Brain Activity Imaging Center using a 12-channel head coil. A forehead pad was used to stabilize the head position. A T1-weighted high-resolution anatomical image was obtained using magnetization-prepared rapid-acquisition gradient-echo sequence (repetition time = 2250 ms; echo time = 3.06 ms; inversion time = 1000; flip angle = 9°; field of view = 256 mm × 256 mm; voxel size = 1 mm × 1 mm × 1 mm).

### Image Analysis

Image and statistical analyses were performed using the statistical parametric mapping package, SPM8^1^ and the VBM8 toolbox^2^, implemented in MATLAB R2012b (MathWorks Inc., Natick, MA, USA). First, image preprocessing was performed using the VBM8 toolbox using default settings. All structural T1 images were segmented into gray matter, white matter, and cerebrospinal fluid using an adaptive maximum a posteriori (AMAP) approach (Rajapakse et al., 1997). Intensity inhomogeneity in the MR image was modeled as slowly varying spatial functions and thus corrected in the AMAP estimation. The segmented images were then used for a partial volume estimation using a simple model with mixed tissue types to improve segmentation (Tohka et al., 2004). Furthermore, a spatially adaptive non-local means denoising filter was applied to deal with spatially varying noise levels (Manjón et al., 2010). A Markov Random Field cleanup was used to improve the image quality. The gray and white matter images in native space were subsequently normalized to the standard stereotactic space defined by the Montreal Neurological Institute using the Diffeomorphic Anatomical Registration using the Exponentiated Lie Algebra algorithm approach (Ashburner, 2007). We used predefined templates provided in the VBM8 toolbox that were derived from 550 healthy brains from the IXI-database^3^. The resulting normalized images of the gray matter and white matter were modulated using Jacobian determinants with non-linear warping only (i.e., m0 image in the VBM8 outputs) to exclude the effect of total intracranial volume. Finally, the normalized modulated images of the gray matter and white matter were resampled to a resolution of 1.5 mm × 1.5 mm × 1.5 mm and smoothed using a 12 mm full-width at half-maximum isotropic Gaussian kernel to compensate for anatomical variability among participants.

To identify the brain regions associated with the Eyes Test score, we performed a multiple regression analysis using test scores as the effect-of-interest independent variable, and sex, age, and full-scale intelligence quotient (IQ; as measured by the Wechsler Adult Intelligence Scale, Third Edition) as effect-of-no-interest covariates. The positive relationship between the Eyes Test and the volume of the gray matter and white matter was tested using T-statistics. Voxels were deemed to be statistically significant if they reached the extent threshold of *p* < 0.05, family-wise error corrected for multiple comparisons determined by Monte Carlo simulations using the AlphaSim function of DPABI software^4^, with the height threshold of *p* < 0.01 (uncorrected). For the analysis of gray matter volumes, we selected the dmPFC, TPJ, amygdala, IFG, and precuneus as the regions of interest (ROIs), which showed activation during performance of the Eyes Test across multiple studies and appear to be involved in TOM, as described in the Introduction. For these regions, we performed small volume correction using the AlphaSim function by constructing anatomical masks of 12-mm radius sphere centered on coordinates taken from previous studies. Information on coordinates was derived from Adams et al. (2010), which reported significant activation during the Asian version of the Eyes Test, in the left dmPFC (x−4, y16, z56), bilateral TPJ (x−48, y−48, z16; x52, y−48, z14), and bilateral IFG (x−54, y32, z−4; x58 y30 z6). Information on the bilateral amygdala (x−26, y−11, z−7; x20 y−8 z−7) and bilateral precuneus (x0, y−47, z59), which was not reported in Adams et al. (2010), was derived from Baron-Cohen et al. (1999). We did not select ROIs for the analysis of white matter volumes because information on these volumes was lacking. Other areas were corrected for the entire brain volume. For exploratory purposes, we tested the interaction between the Eyes Test score and other variables (i.e., sex, age, and full-scale IQ) by adding the interactoin term to the above regression model, although we did not make any specific predictions.

The relationship between the gray/white matter volume and the Eyes Test was illustrated by plotting the gray/white matter values extracted at the peak voxels against test scores after adjusting for the effects-of-no-interest by regressing out sex-, age-, and IQ-related variance.

## Results

### The Eyes Test Score

The psychological rating scores and their correlations are listed in Table 1. The Eyes Test score (mean ± *SD*, 26.7 ± 2.9) was comparable to that obtained in a Japanese sample in a previous study (*t*-test, *p* > 0.1; Adams et al., 2010). Correlation analyses showed no significant correlations between the Eyes Test score and any other variable (sex, age, or full-scale IQ; *p* > 0.1).

**Table 1:** **Eyes Test scores and demographic data, and their correlations**.

Variable	*M/n*	*SD*	Correlation		
			1	2^a^	3
1 Eyes test	26.7	2.9
2 Sex (female:male)	26:25		0.17
3 Age	22.5	4.5	0.16	−0.07
4 Full-scale intelligence quotient	121.4	8.5	0.05	−0.09	−0.03

*^a^Sex is dummy-coded as female (1) or male (0)*.

### Gray Matter Volume Association with the Eyes Test Score

**Figure 1:**
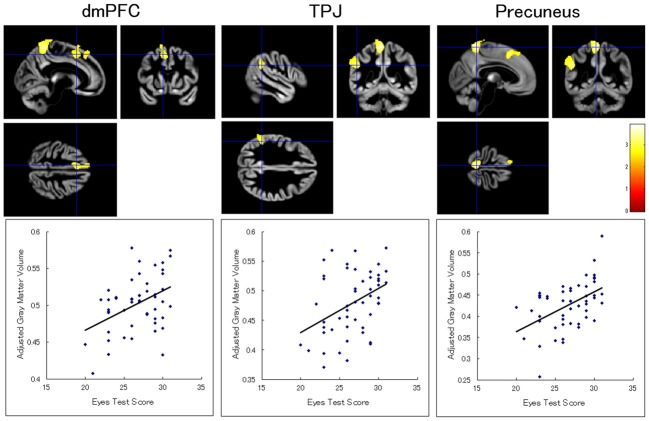
**Brain regions showing a significant positive association between Eyes Test score and gray matter volume.** (Top) Statistical parametric maps showing significant associations with the Eyes Test scores in the dorsomedial prefrontal cortex (dmPFC), temporoparietal junction (TPJ), and precuneus in the left hemisphere. The height threshold is set at an uncorrected *p* < 0.01. The areas are overlaid on the anatomical magnetic resonance images of the mean gray matter of participants involved in the study. The blue crosses indicate the locations of the peak voxels. The red–yellow color scale represents the *T*-value. (Bottom) Scatterplots of the adjusted gray matter volumes as a function of the Eyes Test scores at the peak voxels.

Our ROI analyses revealed significant positive relationships between the Eyes Test score and gray matter volumes in the dmPFC, inferior parietal lobule, and precuneus in the left hemisphere (Table 2; Figure 1). Analyses for ROIs and the whole brain did not show significant association with the Eyes Test score in any other brain regions. We conducted exploratory analyses for the interaction between the Eyes Test score and several other variables (sex, age, and full-scale IQ), but found no significant cluster either in our ROIs or in other brain regions.

**Table 2:** **Brain regions that exhibited a significant positive association. between Eyes Test score and gray matter volume**.

Side	Region	BA	Coordinates			*T*-value	Cluster size (voxels)
			*x*	*y*	*z*
L	Dorsomedial prefrontal cortex	6	−9	14	52	3.27	686
L	Inferior parietal lobule	48	−51	−48	28	3.08	149
L	Precuneus	5	−5	49	66	3.91	453

*BA, Brodmann’s area; L, left. p < 0.05 cluster-level family-wise error-corrected for multiple comparisons within each region of interest (ROI; k > 99 voxels)*.

### White Matter Volume Association with the Eyes Test Score

**Figure 2:**
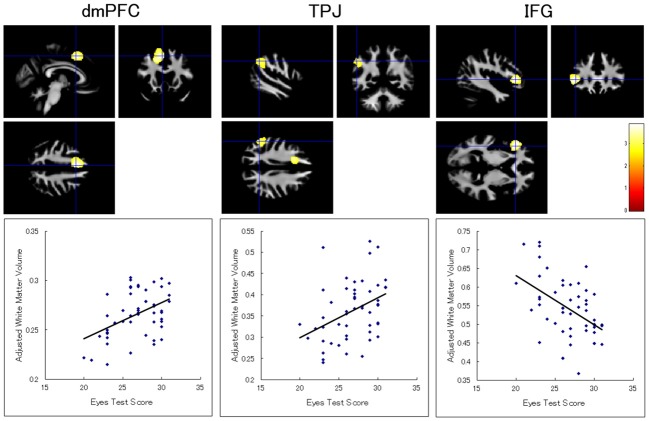
**Brain regions showing a significant association between Eyes Test score and white matter volume.** (Top) Statistical parametric maps showing a significant association with the Eyes Test score in the dmPFC, TPJ, and inferior frontal gyrus (IFG) in the left hemisphere. The height threshold is set at an uncorrected *p* < 0.01. The areas are overlaid on the anatomical magnetic resonance images of the mean white matter of participants involved in the study. The blue crosses indicate the locations of the peak voxels. The red–yellow color scale represents the *T*-value. (Bottom) Scatterplots of the adjusted white matter volumes as a function of the Eyes Test scores at the peak voxels.

There was no white matter region showing a significant association with the Eyes Test score. There was also no significant cluster with respect to the interaction between the Eyes Test score and any other variable (sex, age, and full-scale IQ). When we analyzed the data without the extent threshold, for descriptive purposes, the results showed that the Eyes Test score was positively associated with the white matter volumes in the left dmPFC (x−5, y15, z48; *T*_(46)_ = 3.64; 2306 voxels) and inferior parietal lobule (x−51, y−52, z37; *T*_(46)_ = 2.99; 1027 voxels), and negatively associated with the white matter volume in the left IFG (x−42, y29, z1; *T*_(46)_ = 3.87; 1393 voxels); all of these regions were adjacent to our ROIs (Figure 2).

## Discussion

Our results revealed that the Eyes Test score was positively associated with the gray matter volumes in the dmPFC, inferior parietal lobule (i.e., TPJ), and precuneus in the left hemisphere in normal participants. This result is partly consistent with that of a previous study reporting that the cluster value of gray matter volume in the dmPFC, which showed differences between schizophrenia and control groups, had a positive correlation with the Eyes Test in the normal group (Hirao et al., 2008). Our analysis clearly showed this effect using voxel-by-voxel analysis, and additionally indicated involvement of the TPJ and precuneus. Methodological differences may account for the disparity between these results. Specifically, we used the Asian version of the Eyes Test for Japanese participants, while the previous study used the original Caucasian version to test Japanese participants (Hirao et al., 2008). A previous functional MRI study reported greater behavioral performance and stronger brain activity when the test was composed of stimuli that were culturally similar to the participants (Adams et al., 2010). These data suggest that our culturally optimized test was more sensitive in testing the ability to read the mind in the eyes and its relationship to brain structures. Our results are also consistent with those of all previous functional MRI studies reporting activation of the dmPFC and TPJ while performing the Eyes Test (e.g., Baron-Cohen et al., 1999). Activation of the precuneus was also reported by several functional MRI studies testing the Eyes Test (e.g., Baron-Cohen et al., 1999). Our results extend the literature by indicating a correspondence among the processing of reading the mind in the eyes, functional activation, and gray matter structures. To the best of our knowledge, our study is the first to show that the ability to read the mind in others’ eyes is associated with gray matter volumes in these fronto-temporoparietal regions.

Our results also showed trends toward the Eyes Test score being positively associated with the white matter volumes in the left dmPFC and TPJ and negatively associated with the white matter volume in the left IFG. These regions were adjacent to our gray matter ROIs, which were derived from a previous functional MRI study (Adams et al., 2010). Our results are consistent with several previous structural MRI studies showing that individual differences in cognitive abilities are associated not only with gray matter volumes but also with while matter volumes (e.g., Spencer et al., 2006; for a review, see Bolandzadeh et al., 2012). These data suggest that the consistent ability to read the mind in the eyes is associated with the volumes of both gray and white matter in the fronto-temporoparietal regions.

Our results showed that the Eyes Test score was more obviously related to brain structures in the left hemisphere than to those of the right hemisphere. This appears to be consistent with the result of a previous meta-analysis of functional MRI studies that showed that the left TPJ exhibited substantially more activation while participants performed the Eyes Test than did the right TPJ (Schurz et al., 2014). This result is also in line with a previous neuropsychological finding that performance was impaired on story- and video-based TOM tasks in patients with damage to the left TPJ (Samson et al., 2004). Another neuropsychological study that conducted whole-brain lesion symptom mapping also showed that damage in the left, but not the right, IFG was involved in impaired Eyes Test performance (Dal Monte et al., 2014). Together with these data, our results suggest that the ability to read the mind in the eyes relies more on representations in the left, than the right, fronto-temporoparietal regions.

Our finding that the structural neural substrates of the ability to read the mind in the eyes reside in certain brain regions, including the dmPFC and TPJ, has theoretical implications. Debate remains as to whether the ability to read the mind in the eyes could be accounted for by other general cognitive abilities, such as executive function. Behavioral and neuropsychological evidences are mixed: some studies reported a relationship between performance on the Eyes Test and executive function (e.g., Bull et al., 2008; Dal Monte et al., 2014), whereas other studies did not (e.g., Havet-Thomassin et al., 2006; Ahmed and Stephen Miller, 2011). In contrast, several structural MRI studies investigated the structural neural substrates of executive function and consistently reported that brain regions different from those we found in this study were related to the performance of executive function tests, such as the lateral prefrontal cortex (e.g., Newman et al., 2007; Burzynska et al., 2012). Together with these data, our results suggest that the ability to read the mind in the eyes has, at least partially, specific structural neural substrates dissociable from those for executive functions.

Our results also have practical implications. The MRI-based assessment of the ability to read the minds of others in their eyes may be utilized for clinical purposes. Several behavioral studies have reported that performance on the Eyes Test is impaired in individuals with neuropsychiatric conditions, such as autism spectrum disorders and schizophrenia (e.g., Baron-Cohen et al., 1997). It may be possible to predict such behavioral impairments, and to facilitate objective diagnosis of these psychiatric diseases, using structural MRI.

Although our results provide evidence for the existence of structural neural substrates of reading the mind in the eyes, they do not indicate that the ability is unchangeable. On the contrary, a previous structural MRI study has found that training with meditation changed the cortical thickness of brain regions, including the TPJ (Holzel et al., 2011; Pickut et al., 2013). This finding is consistent with that of a study showing that meditation training increased Eyes Test scores (Mascaro et al., 2013). Together with these findings, our results suggest the possibility that psychological training that effectively modifies the brain structures in the fronto-temporoparietal regions may enhance the ability to read the mind in the eyes.

Several limitations of this study should be acknowledged. First, although some brain regions were found to be significantly associated with the Eyes Test score in this study, several previous functional MRI studies found that other brain regions, such as the amygdala, were activated during the Eyes Test (e.g., Baron-Cohen et al., 1999). This discrepancy may be explained by methodological differences among the studies, for example with respect to measurement of hemodynamic responses vs. gray matter volumes. Alternatively, it may be that our small sample size lacked sufficient power to detect other brain regions. Future studies with larger samples may be necessary to clarify the involvement of other brain regions.

Second, although our analysis indicated the involvement of certain brain regions in mind reading via the eyes, specific functional correlates of these regions remain unknown. Debate also remains in the literature of functional neuroimaging studies (cf. Van Overwalle and Baetens, 2009). For example, it has been proposed that the dmPFC and TPJ might be involved in inferring mental states and gathering important cues, respectively, (Gallagher and Frith, 2003) and that the dmPFC and TPJ might subserve the inference about enduring mental traits and transient mental states, respectively (Van Overwalle, 2009). It may be possible that function-structure relationships are different across the dmPFC and TPJ in a similar way. Future research investigating the association between the structures in these regions and more specific cognitive functions related to reading the mind in the eyes would be promising.

In conclusion, our investigation using structural MRI revealed that Eyes Test scores were positively associated with gray matter volumes in the dmPFC, TPJ, and precuneus in the left hemisphere. The results also showed trends that the Eyes Test scores were positively associated with the white matter volumes in the left dmPFC and TPJ and negatively associated with the white matter volume in the left IFG. These findings suggest that the fronto-temporoparietal network is the structural neural substrate of the ability to read the mind in the eyes.

## Author Contributions

WS, TK, SU, RS, YK, SY, and MT conceived and designed the experiments and wrote the article. WS, TK, SU, RS, YK, and SY performed the experiments. WS and TK analyzed the data.

## Funding

This study was supported by funds from the Japan Society for the Promotion of Science Funding Program for Next Generation World-Leading Researchers (LZ008). The authors declare no competing financial or other interests.

## Conflict of Interest Statement

The authors declare that the research was conducted in the absence of any commercial or financial relationships that could be construed as a potential conflict of interest.

## Footnotes

^1^http://www.fil.ion.ucl.ac.uk/spm

^2^http://dbm.neuro.uni-jena.de

^3^http://www.brain-development.org

^4^http://rfmri.org/dpabi
